# Characteristics, predictors and outcomes of new-onset QT prolongation in sepsis: a multicenter retrospective study

**DOI:** 10.1186/s13054-024-04879-2

**Published:** 2024-04-09

**Authors:** Weizhuo Liu, Rongjiao Shao, Shen Zhang, Lei Jin, Rongchen Liu, Peidong Chen, Jintao Hu, Haocheng Ma, Bangwei Wu, Weiguo Liang, Xinping Luo, Jian Li, Weiming Chen, Nanqing Xiong, Bin He

**Affiliations:** 1 Department of Critical Care Medicine, Shanghai Chest Hospital, Shanghai Jiaotong University School of Medicine, 241 Huaihaixi Road, Shanghai, 200030 China; 2 Centre for Cardiopulmonary Translational Medicine, Shanghai Chest Hospital, Shanghai Jiaotong University School of Medicine, Shanghai, China; 3 Department of Cardiology, Huashan Hospital Fudan University, 12 Wulumuqizhong Road, Shanghai, 200030 China; 4 Department of Infectious Diseases, Jing’an District Central Hospital of Shanghai, Fudan University, Shanghai, China; 5 Department of Cardiology, People’s Hospital of Qiubei, Putan Road in Jinping Town, Qiubei, 663200 Yunnan China; 6 Department of Cardiology, First Affiliated Hospital of Kunming Medical University, Kunming, Yunnan China

**Keywords:** New-onset QT prolongation, Sepsis, Ventricular arrhythmia

## Abstract

**Background:**

Sepsis-induced myocardial injury is a serious complication of sepsis. QT prolongation is a proarrhythmic state which reflects myocardial injury in a group of heterogeneous disorders. However, the study on the clinical value of QT prolongation in sepsis is limited.

**Methods:**

We aimed to investigate the clinical characteristics and predictors of new-onset QT prolongation in sepsis and its impact on the outcome in a multicenter retrospective cohort study. Electrocardiographic and clinical data were collected from patients with sepsis from the wards and intensive care units of four centers after exclusion of QT-influencing medications and electrolyte abnormalities. Clinical outcomes were compared between patients with and without QT prolongation (QTc > 450 ms). Multivariate analysis was performed to ascertain whether QT prolongation was an independent predictor for 30-day mortality. The factors predicting QT prolongation in sepsis were also analyzed.

**Results:**

New-onset QT prolongation occurred in 235/1024 (22.9%) patients. The majority demonstrated similar pattern as type 1 long QT syndrome. Patients with QT prolongation had a higher 30-day in-hospital mortality (*P* < 0.001), which was also associated with increased tachyarrhythmias including paroxysmal atrial fibrillation or tachycardia (*P* < 0.001) and ventricular arrhythmia (*P* < 0.001) during hospitalization. QT prolongation independently predicted 30-day mortality (*P* = 0.044) after multivariate analysis. History of coronary artery disease (*P* = 0.001), septic shock (*P* = 0.008), acute respiratory (*P* < 0.001), heart (*P* = 0.021) and renal dysfunction (*P* = 0.013) were independent predictors of QT prolongation in sepsis.

**Conclusions:**

New-onset QT prolongation in sepsis was associated with increased mortality as well as atrial and ventricular arrhythmias, which was predicted by disease severity and organ dysfunction.

**Graphical abstract:**

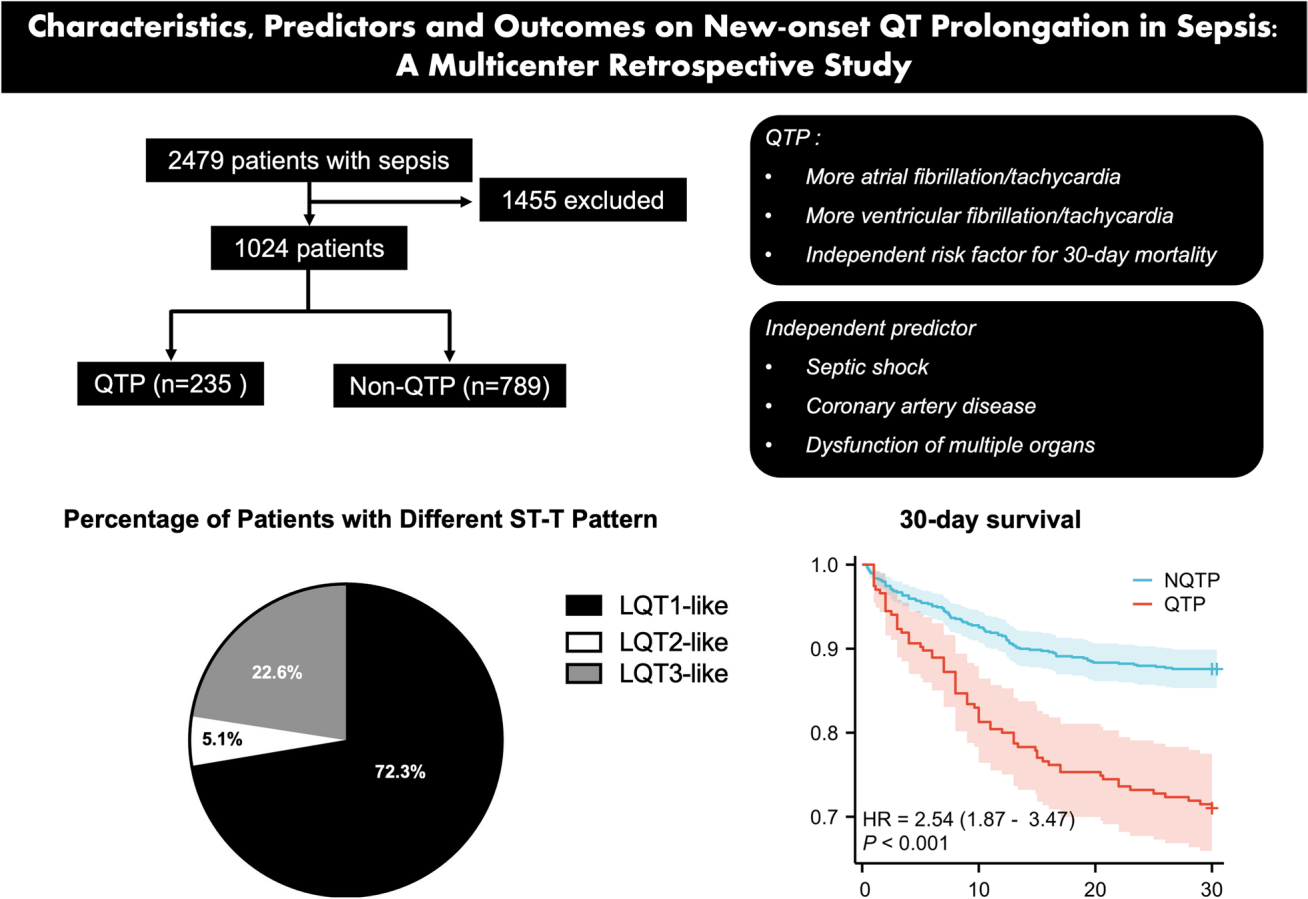

**Supplementary Information:**

The online version contains supplementary material available at 10.1186/s13054-024-04879-2.

## Introduction

Myocardial injury is a serious complication of sepsis which can manifest as decreased myocardial contractility, weakened response to cardiac preload, atrial and ventricular arrhythmias [1, 2]. Prolongation of QT interval is a proarrhythmic status reflecting the increased duration of ventricular activation, which can lead to fatal ventricular arrhythmias such as polymorphic ventricular tachycardia (VT) including Torsade de Pointes VT and ventricular fibrillation (VF) [3, 4].

In addition to various types of congenital long QT syndrome, acquired QT prolongation (QTP) due to heterogeneous etiology, including myocardial ischemia, specific medications and abnormal electrolytes in different diseases, has been well described [5, 6]. QT prolongation has been considered as a consequence of myocardial injury in the setting of sepsis [7]. However, limited data have been shown with respect to the clinical value and related factors.

In this multicenter retrospective cohort study, we aimed to investigate the proportion, electrocardiogram (ECG) characteristics, clinical relevance and the risk factors of new-onset QT prolongation in patients with sepsis.

## Methods

### Study population

In-hospital patients diagnosed as sepsis according to the criteria of sepsis 3.0 [1] from the wards and intensive care units of four centers from 2018 to 2022, were consecutively enrolled in the study. Exclusion criteria included: (1) Patients without ECG performed at the time of diagnosis of sepsis (within 48 h); (2) history of congenital or other acquired long QT syndrome; (3) regular intake of medications affecting QT interval, e.g., types 1 and 3 antiarrhythmic agents and anti-depression agents (see Additional file 1); (4) administration of QT-influencing antibiotics before electrocardiogram (ECG) test; (5) absolute irregular rhythm, e.g., atrial fibrillation or 2nd degree atrioventricular block and (6) extremely abnormal electrolyte level potentially causing QTP, including serum potassium level < 2.7 mmol/L and serum calcium level < 1.6 mmol/l [8, 9]. Patients with COVID-19 infection were not admitted to these centers during the study period.

### Clinical data collection

ECGs were recorded with a paper speed of 25 mm/s and amplitude of 10 mm/mV and stored in digital version. QT interval was manually measured and corrected with Bazett formula (corrected QT interval (QTc) = QT/RR^1/2^) when heart rate (HR) was less than or equal to 110/min, or with Hodges formula QTc = QT + 1.75(HR-60), when HR was over 110/min [10]. According to a previous study, QTP was defined as QTc > 450 ms, while T wave morphology was classified into three types as in the congenital long QT syndromes [11]: “LQT1-like morphology” denoted a long QT interval with broad T waves; “LQT2-like morphology” denoted a long QT interval with notched T waves and “LQT3-like morphology” denoted a long QT interval with small T waves separated from the QRS interval by a long isoelectric ST-segment. In patients with QTP, QT_onset_ and QT_peak_ were defined as the interval from the Q wave to the point at which the T wave departs from the flat portion of the ST-segment, and to the peak of the T wave, respectively [12, 13]. T wave duration (TWD)/QT and T_peak_-T_end_/QT could be, thereafter, calculated based on the above data. New-onset QTP was defined as prolongation of QT interval at the time of sepsis diagnosis without history of QTP or previously recorded ECG showing QTc > 450 ms. All the ECGs were re-analyzed by two independent cardiologists (R.L. and H.M.) with average values as results. The interpretation was carried out by clinicians without knowing the other data including the outcome of the patients.

The clinical variables were retrieved from the hospital information system. The baseline data including age, sex, body mass index, body surface area, history of chronic diseases and recent surgery were collected. Charlson score was used to assess the general comorbidity conditions. Structural heart diseases included ischemic cardiomyopathy, severe valvular heart diseases (with or without surgical repair) and non-ischemic (dilated, hypertrophic, restrictive or arrhythmogenic) cardiomyopathies. Renal insufficiency was defined as stage 4+ chronic kidney disease.

The complications including septic shock and dysfunction of organs were evaluated at the diagnosis time. Septic shock was defined as a state of acute circulatory failure due to sepsis manifest as hypotension (mean arterial pressure < 65 mmHg) and raised lactate level, requiring adequate fluid resuscitation and/or vasopressor therapy [14]. Acute heart failure was defined as rapid or gradual onset of symptoms and/or signs and imaging evidence of heart failure requiring urgent treatment, with an NT-proBNP level > 300 pg/ml [15]. Acute renal failure was defined as an increase in serum creatinine by ≥ 0.3 mg/dl (26.5 µmol/l) within 48 h, or increase in creatinine to ≥ 1.5 times baseline occurring within the prior 7 days, or urine volume < 0.5 ml/kg/h for 6 h [16]. Acute respiratory failure was defined as new or acutely worsening respiratory symptoms following a known clinical insult lasting less than a week and PaO_2_/FiO_2_ less than or equal to 300 mmHg while receiving additional oxygen by a standard facial mask [17]. Acute liver failure was defined in the study as an acute liver function deterioration with or without preexisting liver diseases, manifesting as jaundice, coagulopathy, ascites and/or hepatic encephalopathy within 4 weeks [18, 19]. The results of laboratory tests were also recorded. Sequential organ failure assessment (SOFA) and quick sequential organ failure assessment (qSOFA) scores could, therefore, be calculated based on the data [14].

During hospitalization, any episodes of atrial fibrillation (AF), atrial tachyarrhythmia (AT), VT and VF demonstrated in ECG tracing, Holter or telemetry were counted as tachyarrhythmias. AF was defined as the absence of P wave replaced by fibrillatory atrial activities with irregular R–R intervals. AT was defined as regular atrial rhythm, including focal and macro-reentrant atrial tachycardia (flutter) but not junctional tachycardia, at a constant rate of > 100/min with discrete P or F waves and atrial activation sequences originating outside of the sinus node [20]. Ventricular tachycardia was defined as more than three consecutive ventricular beats with a rate > 100/min, independent from atrial and AV nodal conduction. Ventricular fibrillation was defined as chaotic rhythm with undulations that are irregular in timing and morphology, without discrete QRS complexes on the surface ECG [21]. The study was approved by the Ethics Committee of Shanghai Chest Hospital, Shanghai Jiaotong University School of Medicine (IRB No. KS22024).

### Statistical analysis

Clinical variables were expressed as a percentage (%) for categorical variables, mean with standard deviation for normally distributed continuous variables and median with interquartile rate for discontinuous variables. To compare categorical variables, Chi-square test or Fisher’s exact test was used; to compare continuous variables, an unpaired two-tailed *t*-test or Mann–Whitney *U*-test was used. In laboratory verification, data were presented as the mean ± SEM at least three duplications of different samples. Student’s *t*-test was applied for analysis between two groups, and one-way analysis of variance was used for comparisons between multiple groups. Multivariate analysis was performed to ascertain whether QTP was an independent predictor for prognosis and to explore the predictors of QTP using logistics regression, which adjusted for all variables showing statistical differences in univariate analysis and of vital importance in clinical practice. Correlated variables with collinearity were not repetitively taken into analysis in one model. Multiple regression models were established for further validation. Statistical analysis was performed with Stata 16.0 software. GraphPad Prism 7.0 (GraphPad Software Inc., USA) was used to analyze and illustrate the data. Differences with p-values < 0.05 were considered statistically significant.

## Results

### Clinical characteristics and the incidence of new-onset QTP

**Fig. 1:**
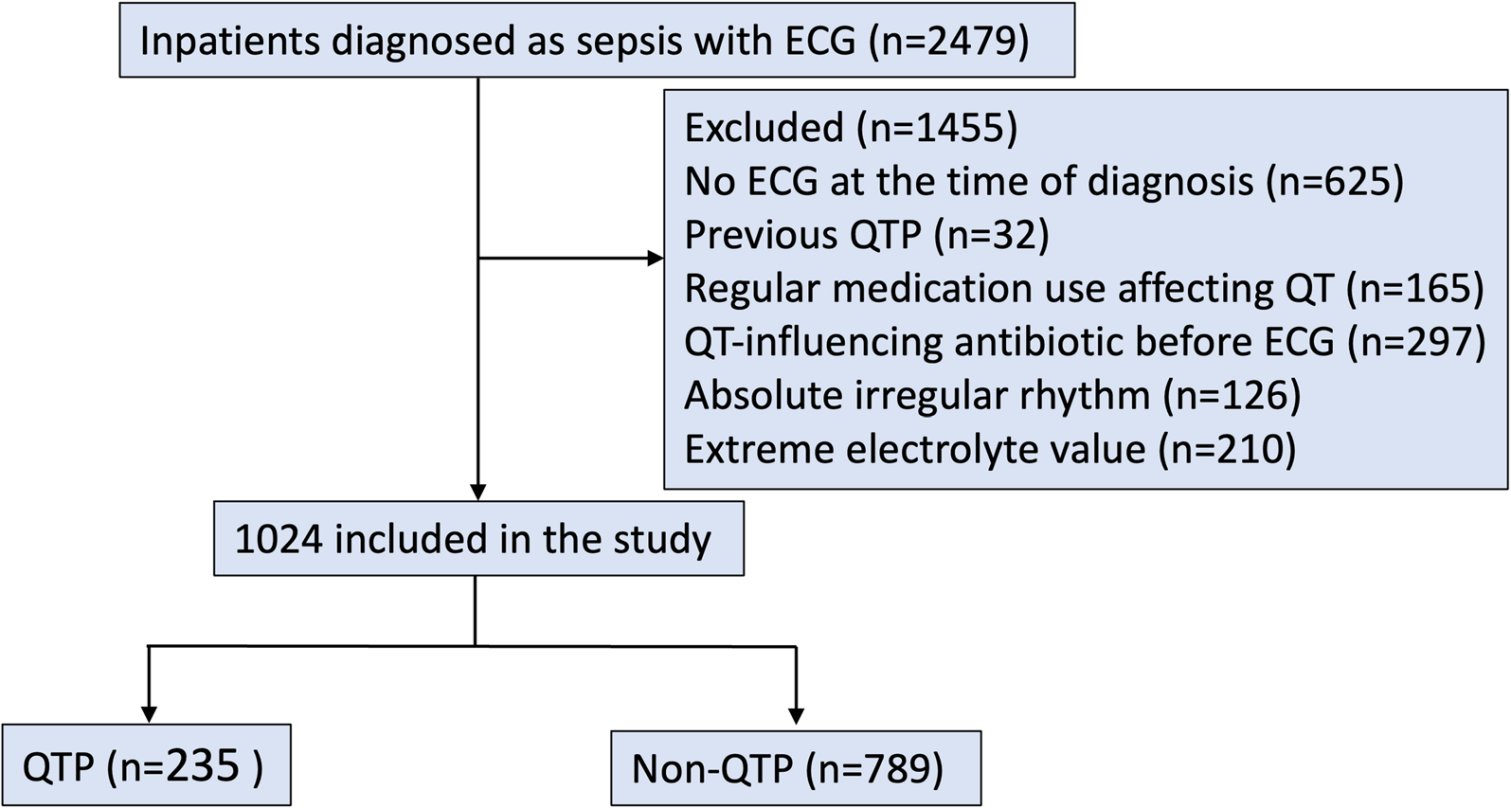
Flowchart of the patient inclusion in this study. *QTP* QT prolongation

**Fig. 2:**
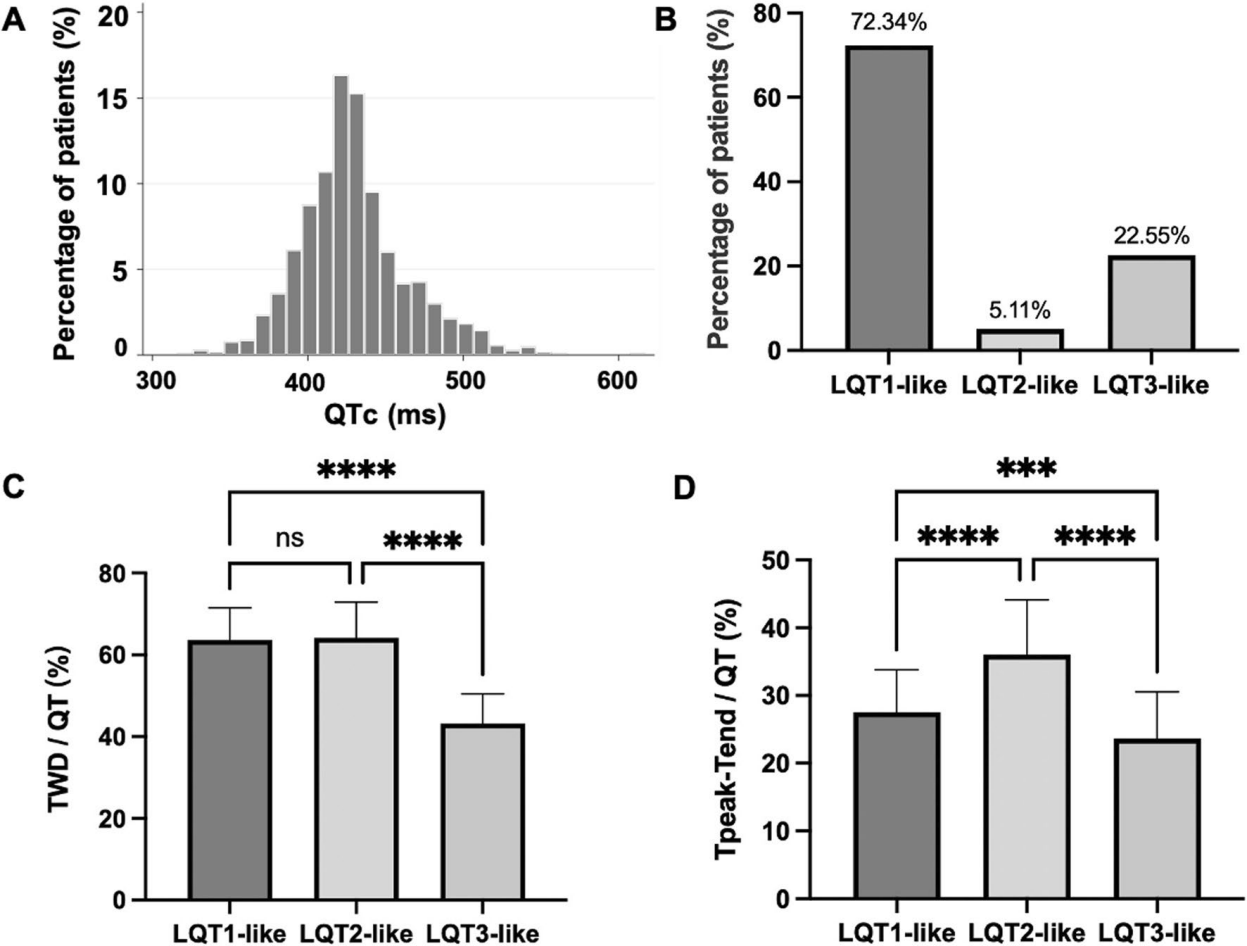
**A** Distribution of the corrected QTc in the whole study cohort. **B** Proportion of different QT morphologies in patients with QT prolongation. **C** T wave duration/QT for each LQT-like phenotype. **D** *T*_peak_-*T*_end_/QT for each LQT-like phenotype. See text for discussion. *LQT* long QT and *QTc* corrected QT interval

A total of 2479 patients matched the diagnosis of sepsis during the study period. After exclusion of QTP history, QT-influencing medications, electrolyte abnormalities, irregular rhythm and lack of timely ECGs, a total number of 1024 subjects, including 382 (37.3%) patients from the intensive care units, were enrolled and divided into QTP and non-QTP groups (Fig. 1). The distribution of QTc is shown in Fig. 2A. The mean QTc in the whole cohort was 430.1 ± 35.3 ms, in which QTP occurred in 235 (22.9%) individuals.

QTP was found statistically related to higher age (*P* = 0.002), lower body surface area (*P* = 0.013), hypertension (*P* = 0.044), coronary artery disease (*P* < 0.001), structural heart disease (*P* < 0.001), renal insufficiency (*P* = 0.002) and chronic occlusive pulmonary disease (*P* < 0.001). QTP was associated with septic shock (*P* < 0.001), multiple organ dysfunction (all *P* < 0.01) and a higher SOFA (*P* < 0.001) and qSOFA score (*P* < 0.001). Patients with QTP had an increased 30-day mortality (*P* < 0.001), but without difference in hospital stay (Table 1).

**Table 1:** Baseline and disease characteristics in patients with and without QTP

	Non-QTP (*n* = 789)	QTP^a^ (*n* = 235)	*P* value
*Patient characteristics*			
Age, years	59.9 ± 16.2	63.6 ± 15.3	0.002
Sex, female, %	251 (31.8)	81 (34.5)	0.445
Body mass index (kg/m^2^)	23.25 ± 3.55	22.97 ± 3.46	0.292
Body surface area (m^2^)	1.73 ± 0.17	1.69 ± 0.19	0.013
*Baseline comorbidities*			
Hypertension, %	215 (27.25)	80 (34.04)	0.044
Diabetes mellitus, %	170 (21.55)	53 (22.55)	0.743
Coronary artery disease, %	44 (5.58)	37 (15.74)	< 0.001
Structural heart disease, %	38 (4.82)	30 (12.77)	< 0.001
Congenital heart disease, %	12 (1.52)	2 (0.85)	0.438
Renal insufficiency, %	35 (4.44)	23 (9.79)	0.002
COPD^b^, %	35 (4.44)	35 (14.89)	< 0.001
Liver cirrhosis, %	23 (2.92)	10 (4.26)	0.307
Charlson score	1 [0, 2]	2 [1, 3]	< 0.001
Recent^c^ surgery, %	129 (16.35)	42 (17.87)	0.583
Recent^c^ chemo/immunotherapy, %	59 (7.48)	11 (4.68)	0.136
*Clinical characteristics*			
Severe sepsis/shock, %	159 (20.15)	110 (46.80)	< 0.001
qSOFA^d^ score	1 [1, 2]	2 [1, 2]	< 0.001
SOFA^e^ score	3 [2, 6]	6 [3, 8]	< 0.001
Acute heart failure, %	68 (8.62)	70 (29.79)	< 0.001
Acute renal failure, %	127 (16.10)	95 (40.43)	< 0.001
Acute respiratory failure, %	124 (15.72)	114 (48.51)	< 0.001
Acute liver failure, %	47 (5.96)	26 (11.06)	0.008
*Laboratory results*			
White blood cell count, *10^9^/L	10.38 ± 8.70	13.20 ± 18.57	0.001
Neutrophil, %	75.37 ± 16.42	82.21 ± 31.64	< 0.001
Hemoglobin, g/L	106.84 ± 24.93	107.12 ± 27.22	0.880
C-reactive protein, mg/L	75.33 ± 72.59	92.14 ± 78.56	0.018
Procalcitonin, ng/mL	10.39 ± 42.88	12.39 ± 23.50	0.493
Lactate level	2.84 ± 2.33	4.03 ± 3.12	< 0.001
Pro-BNP, pg/mL	585.2 [159.4, 2520.0]	1383.7 [458.4, 4945.0]	< 0.001
Troponin T, ng/mL	0.136 ± 0.823	0.141 ± 0.758	0.958
Creatinine, μmoI/L	107.75 ± 130.77	168.43 ± 210.62	< 0.001
Bilirubin, mmol/L	22.24 ± 50.94	27.70 ± 56.72	0.161
Alanine transaminase, U/L	58.70 ± 191.20	83.94 ± 305.19	0.127
Albumin, g/L	33.74 ± 6.61	32.72 ± 6.45	0.038
Serum potassium, mmol/L	3.86 ± 0.57	3.89 ± 0.72	0.457
Serum calcium, mmol/L	2.312 ± 0.19	2.12 ± 0.20	0.964
*Etiology*			
Positive blood culture	353 (44.74)	84 (35.74)	0.014
Gram-positive bacteria, %	139 (39.38)	23 (27.38)	0.041
Gram-negative bacteria, %	200 (56.66)	58 (69.05)	0.038
Non-bacteria, %	20 (5.67)	8 (9.52)	0.194
*Portal of entry of the infection*			
Lungs	306 (38.78)	122 (51.91)	0.001
Abdominal	178 (22.56)	58 (24.68)	0.498
Cardiovascular	52 (6.59)	13 (5.53)	0.559
Central nervous system	71 (9.00)	17 (7.23)	0.314
Urinary	157 (19.90)	42 (17.87)	0.491
Catheter-related	33 (4.18)	10 (4.26)	0.961
Others	87 (11.03)	25 (10.64)	0.867
Unknown	79 (10.01)	15 (6.38)	0.091
*Treatment*			
Antibiotic use	788 (99.87)	233 (99.15)	0.361
Vasoactive drugs	189 (23.95)	101 (42.98)	< 0.001
Mechanical ventilation	235 (29.78)	119 (50.64)	< 0.001
Tracheal intubation	139 (17.62)	89 (37.87)	< 0.001
Fluids infused	206 (26.11)	117 (49.79)	< 0.001
Outcome			
In-hospital mortality, %	118 (14.96)	77 (32.77)	< 0.001
Length of hospital stay, days	12.0 [7.0, 19.6]	11.4 [6.6, 19.0]	0.322
30-day mortality, %	98 (12.42)	67 (28.51)	< 0.001

^a^QTP = QT prolongation

^b^COPD = chronic obstructive pulmonary disease

^c^Within 30 days

^d^qSOFA = quick sequential organ failure assessment

^e^SOFA = sequential organ failure assessment

### Description and comparison of ECG parameters

In terms of morphology, the majority of the patients (72.3%) had an LQT-1-like T wave, followed by LQT-3- (22.6%) and LQT-2-like (5.1%) configuration (Fig. 2A–B). TWD/QT and *T*_peak_-*T*_end_/QT were 59.1 ± 11.5% and 27.1 ± 7.0%, respectively. TWD/QT was lower in LQT-3 (43.3 ± 7.2%) than in LQT-1 (63.6 ± 7.9%) and LQT-2 (64.2 ± 8.7%) pattern (both *P* < 0.001), without difference in the latter two (LQT-1 vs. LQT-2, *P* > 0.999). *T*_peak_-*T*_end_/QT was highest in LQT-2-like pattern (36.4 ± 8.2%), followed by LQT-1- (27.6 ± 6.2%) and LQT-3-like pattern (23.7 ± 6.9%) with statistical significance (all *P* < 0.001) (Fig. 2C–D). Patients with new-onset QTP had a higher sinus heart rate (97.9 ± 24.4 bpm vs. 90.0 ± 21.3 bpm, *P* < 0.001) and a wider QRS width (98.7 ± 23.3 ms vs. 89.8 ± 13.0 ms, *P* < 0.001) in baseline ECG compared to non-QTP patients (Table 2).

**Table 2:** ECG parameters and arrhythmias during hospitalization in patients with and without QTP

ECG parameters	Non-QTP^a^ (n = 789)	QTP (n = 235)	*P* value
*ECG at diagnosis*			
Heart rate, bpm	90.9 ± 21.3	97.9 ± 24.4	< 0.001
QRS duration, ms	89.8 ± 13.0	98.7 ± 23.3	< 0.001
PR interval, ms	151.9 ± 27.3	151.4 ± 28.0	0.829
QT interval, ms	346.0 ± 42.3	387.5 ± 43.5	< 0.001
Corrected QT interval^b^, ms	415.3 ± 22.0	479.6 ± 25.1	< 0.001
Axis, degree	38 [13, 63]	39 [9, 68]	0.672
RV5 + SV1, MV	2.12 ± 1.13	2.03 ± 1.11	0.296
TWD^c^/QT	–	59.1 ± 11.5	–
*T*_peak_-*T*_end_^d^/QT	–	27.1 ± 7.0	–
U wave, %	0 (0)	9 (3.83)	< 0.001
Atrioventricular block, %	25 (3.17)	11 (4.68)	0.269
Left bundle branch block, %	1 (0.13)	3 (1.28)	0.013
Right bundle branch block, %	36 (4.56)	27 (11.49)	< 0.001
Left ventricular hypertrophy, %	38 (4.82)	16 (6.81)	0.230
Sinus tachycardia, %	234 (29.66)	84 (35.74)	0.077
Sinus bradycardia, %	22 (2.79)	2 (0.85)	0.085
ST-segment depression, %	36 (4.56)	9 (3.83)	0.630
*Arrhythmic events during hospitalization*			
Paroxysmal AF or AT^e^, %	36 (4.56)	31 (13.19)	< 0.001
Frequent PVCs^f^, %	29 (3.58)	19 (8.09)	0.005
VT or VF^g^, %	5 (0.63)	13 (5.53)	< 0.001

^a^QTP = QT prolongation

^b^Corrected with Bazett formula (Corrected QT interval (QTc) = QT/RR^1/2^) when heart rate (HR) was less than or equal to 110/min, or with Hodges formula QTc = QT + 1.75(HR-60), when HR was over 110/min

^c^TWD = T wave duration

^d^Defined as the time interval from the peak to the end of the T wave

^e^AF = atrial fibrillation and AT = atrial tachyarrhythmia

^f^PVC = premature ventricular contraction

^g^VT = ventricular tachycardia and VF = ventricular fibrillation

### Relationship between new-onset QTP and clinical outcome in sepsis

**Fig. 3:**
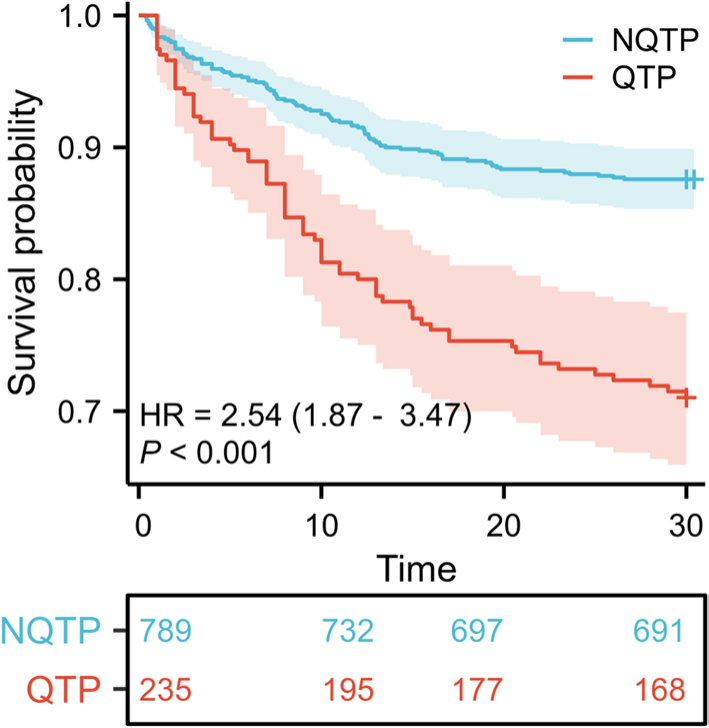
Kaplan–Meier survival curve of the study cohort. Patients with QTP had a higher 30-day mortality rate compared to the controls (both *P* < 0.001). *NQTP* non-QT prolongation and *QTP* QT prolongation

Kaplan–Meier survival curve showed that new-onset QTP was associated with a higher 30-day mortality compared to those without QTP (both *P* < 0.001, Fig. 3). After multivariate analysis adjusted for indicators showing significant difference in univariate analysis, QTP was an independent predictor for 30-day mortality while adjusted for age, body mass index, recent surgery, structural heart disease, Charlson score and SOFA score (Additional file 2: Table S1A–B). In addition, multiple regression models were performed to further validate the relations between QTP and the 30-day mortality (Additional file 2: Table S1C, all *P* < 0.05).

During hospitalization, patients showing QTP had a higher risk for developing tachyarrhythmias, including paroxysmal AF/AT (13.19% vs. 4.56%, *P* < 0.001), VT and VF (5.5% vs. 0.63%, *P* < 0.001). QTc in patients with AF/AT or VT/VF was longer than those without tachyarrhythmias (452.6 ± 39.6 ms, 428.14 ± 34.28, *P* < 0.001). Among patients with QTP, ECG parameters were compared between those with different outcomes during hospitalization, i.e., survived or died. Patients who died within 30 days had a higher heart rate (*P* < 0.001), a higher QTc (*P* = 0.005) and a rightward axis (*P* = 0.002). TWD/QT and *T*_peak_-*T*_end_/QT did not show difference in the two subgroups (Table 3).

**Table 3:** Comparison between QTP patients with different outcomes during hospitalization

ECG parameters	30-day survival (*n* = 168)	30-day mortality (*n* = 67)	*P* value
Heart rate, bpm	93.35 ± 19.62	109.39 ± 30.74	< 0.001
QRS duration, ms	98.16 ± 23.22	99.99 ± 23.61	0.589
PR interval, ms	152.34 (27.82)	149.16 (28.48)	0.434
QT interval, ms	390.90 (41.39)	378.96 (47.63)	0.057
Corrected QT, ms	476.70 (23.32)	486.76 (28.15)	0.005
Axis, degree	34 [3.5, 63.5]	58 [21, 76]	0.002
RV5 + SV1, ms	2.04 (1.03)	2.01 (1.32)	0.816
TWD/QT	59.88 (10.74)	57.02 (13.15)	0.085
*T*_peak_-*T*_end_/QT	27.63 (7.12)	25.85 (6.49)	0.077
U wave	8 (4.76)	1 (1.49)	0.238

### Risk factors for new-onset QTP in sepsis

In multivariate analysis, coronary artery disease (OR 2.51, *P* = 0.001), septic shock (OR  1.71, *P* = 0.008), acute heart failure (OR   1.73, *P* = 0.021), acute kidney injury (OR   1.67, *P* = 0.013) and acute respiratory failure (OR   2.66, *P* < 0.001) were independently associated with QTP (Table 4). In addition, the relationship between QTP and all the five above factors was confirmed in different regression models (Additional file 3: Table S2).

**Table 4:** Multivariate analysis for prediction of new-onset QTP in sepsis

Variable	OR	95% CI for OR		*P* value
		Lower	Upper	
Coronary artery disease, %	2.51	1.46	4.30	0.001
Septic shock, %	1.71	1.15	2.53	0.008
Acute heart failure, %	1.73	1.09	2.75	0.021
Acute renal failure, %	1.67	1.12	2.50	0.013
Acute respiratory failure, %	2.66	1.77	3.99	< 0.001

Adjusted for age, body surface area, hypertension, coronary heart disease, structural heart disease, COPD, septic shock, acute heart failure, acute renal failure, acute respiratory failure and acute liver failure that show significant difference in univariate analysis

*QTP* QT prolongation

## Discussion

In the present study, we have following findings regarding the new-onset QTP in sepsis including the prevalence, ECG characteristics, prognostic value and the risk factors:

1. QTP occurred in 22.9% patients with sepsis in this study. The morphological QT change was variable, mostly manifesting as the prolongation of T wave (LQT-1-like configuration).
2. New-onset QTP independently predicted 30-day mortality in sepsis.
3. Patients with QTP had more atrial and ventricular tachyarrhythmias during hospitalization.
4. QTP was independently predicted by disease severity and the dysfunction of multiple organs.

Acquired QTP was secondary to a group of heterogeneous diseases or pathophysiologic status. The vast majority of acquired LQT syndrome is caused by the drug effect or abnormal electrolyte [22–25]. It is not infrequent to see the phenomenon of QTP in patients with sepsis [26, 27], which is, however, less reported and often explained by the use of antibiotics [28]. In a previous study, 10.5% patients with sepsis had a QTc duration longer than 490 ms without including those treated by QT-influencing medications [29]. In our study, the patients receiving therapy with QT-affecting antibiotics have also been excluded, so were the subjects with extreme values of electrolytes, which can be proven by the similar serum potassium and calcium levels in univariate analysis.

QT interval was composed of the entire duration of ventricular depolarization and the repolarization. The majority of the patients showed T wave prolongation similar to type 1 long QT syndrome, i.e., the prolongation occurred mainly in the T wave duration, which indicated that the decrease in potassium efflux might be an underlying reason [11, 30]. T wave patterns in congenital LQT syndrome (LQT-1/2/3 like) have been used in acquired QTP to describe the morphology. The difference in pattern indicates the functional variation of different ion channels and may lead to different outcomes in specific settings. The majority of QTP in the current study demonstrated an LQT-1-like pattern, suggesting that the downregulation of potassium channel might be an underlying reason for the lengthening of ventricular activation time.

In our study, QTP demonstrated prognostic value as indicated by the Kaplan–Meier curve and multivariate analysis. This observation aligns with the findings of a previous study [29]. For a more robust conclusion, multiple models were used in our study to confirm QTP as the independent predictor for 30-day mortality. QTP has been described as a commonly encountered ECG change in a group of heterogeneous diseases such as myocardial infarction [31], myocarditis [32] and viral infection [33]. It was associated with not only ventricular but also AT/AF despite the exclusion of persistent atrial fibrillation, suggesting the potential increase in general electrical vulnerability. This was further supported by the higher QTc in the patients experiencing those tachyarrhythmias. Considering the consequence of increased fatal arrhythmias, maintaining the serum potassium level at a normal range is of potential benefit due to its effect on potassium efflux.

Organ failure is a marker for deterioration of the disease and the compromised outcome in sepsis [34]. Several models were established in the multivariate analysis for robust evidence supporting the factors as independent predictors for QTP. Dysfunction of multiple organs, i.e., cardiac, respiratory and renal failure, was independent predictors for QTP in our study, indicating the association between the disease severity and the QT interval. We also found that coronary artery disease was a predictor for QTP, which showed similar results to a previous study conducted in cardiac care units [27]. The ratios of TWD/QT and *T*_peak_-*T*_end_/QT did not show significance in patients with a worse prognosis, which was different from the previous study [35].

Additionally, individual differences were observed in the presence of QTP even in similar clinical settings, suggesting that the effect of sepsis on the expression of ion channel proteins could be heterogeneous. This necessitates further investigation.

## Limitations

This work was a retrospective cohort study and needed further validation with a prospective study. Patients without timely ECG were excluded, which may alter the frequency of QTP. Serum magnesium level was not available in part of the subjects. QTP due to hypomagnesemia was, therefore, not excluded.

## Conclusions

In patients with sepsis showing new-onset QTP, the majority of changes in QT morphology was manifested as T wave prolongation. New-onset QTP was associated with worse prognosis and was associated with increased atrial and ventricular arrhythmias. QTP could be predicted by the disease severity and dysfunction of multiple organs.

### Supplementary Information

**Additional file 1**. QT-Prolonging Medications for Exclusion in the Study.

**Additional file 2**. **Table S1**: Predictors of 30-day mortality in patients with sepsis.

**Additional file 3**. **Table S2**: Multiple regression models for validate the risk factors for QTP.

## Data Availability

The datasets used and/or analyzed during the current study are available from the corresponding author on reasonable request.

## Abbreviations

AF: Atrial fibrillation
AT: Atrial tachyarrhythmia
ECG: Electrocardiogram
HR: Heart rate
QTc: Corrected QT interval
QTP: QT prolongation
(q)SOFA: (Quick) sequential organ failure assessment
TWD: T wave duration
VF: Ventricular fibrillation
VT: Ventricular tachycardia

## References

[1] Hollenberg SM, Singer M (2021). Pathophysiology of sepsis-induced cardiomyopathy. Nat Rev Cardiol.

[2] L’Heureux M, Sternberg M, Brath L, Turlington J, Kashiouris MG (2020). Sepsis-induced cardiomyopathy: a comprehensive review. Curr Cardiol Rep.

[3] Schwartz PJ, Crotti L, Insolia R (2012). Long-QT syndrome: from genetics to management. Circ Arrhythm Electrophysiol.

[4] El-Sherif N, Turitto G, Boutjdir M (2018). Acquired long QT syndrome and torsade de pointes. Pacing Clin Electrophysiol.

[5] Rodríguez-Jiménez AE, Cruz-Inerarity H, Negrín-Valdés T, Fardales-Rodríguez R, Chávez-González E (2019). Corrected QT-interval dispersion: an electrocardiographic tool to predict recurrence of myocardial infarction. MEDICC Rev.

[6] Ahmed R, Kiya F, Kitano K, Takagi H, Hashiba K (1987). Effects of combined changes in serum calcium and potassium on QT interval. A study by Holter electrocardiographic monitoring during hemodialysis. Jpn Heart J.

[7] Varriale P, Ramaprasad S (1995). Septic cardiomyopathy as a cause of long QT syndrome. J Electrocardiol.

[8] Krogager ML, Kragholm K, Skals RK, Mortensen RN, Polcwiartek C, Graff C (2019). The relationship between serum potassium concentrations and electrocardiographic characteristics in 163,547 individuals from primary care. J Electrocardiol.

[9] Marriott H, Marriott H (1988). Miscellaneous conditions-hypokalemia. Practical electrocardiography.

[10] Luo S, Michler K, Johnston P, Macfarlane PW (2004). A comparison of commonly used QT correction formulae: the effect of heart rate on the QTc of normal ECGs. J Electrocardiol.

[11] Topilski I, Rogowski O, Rosso R, Justo D, Copperman Y, Glikson M, Belhassen B, Hochenberg M, Viskin S (2007). The morphology of the QT interval predicts torsade de pointes during acquired bradyarrhythmias. J Am Coll Cardiol.

[12] Tardo DT, Peck M, Subbiah RN, Vandenberg JI, Hill AP (2023). The diagnostic role of T wave morphology biomarkers in congenital and acquired long QT syndrome: a systematic review. Ann Noninvasive Electrocardiol.

[13] Moss AJ, Zareba W, Benhorin J, Locati EH, Hall WJ, Robinson JL, Schwartz PJ, Towbin JA, Vincent GM, Lehmann MH (1995). ECG T-wave patterns in genetically distinct forms of the hereditary long QT syndrome. Circulation.

[14] Singer M, Deutschman CS, Seymour CW, Shankar-Hari M, Annane D, Bauer M (2016). The third international consensus definitions for sepsis and septic shock (sepsis-3). JAMA.

[15] McDonagh TA, Metra M, Adamo M, Gardner RS, Baumbach A, Böhm M (2021). 2021 ESC Guidelines for the diagnosis and treatment of acute and chronic heart failure. Eur Heart J.

[16] Group KDIGOKAKIW (2012). KDIGO clinical practice guideline for acute kidney injury. Kidney Int.

[17] Fernández Ceballos I, Steinberg E, Ems J, Nuñez Silveira JM, Madorno M, Carboni Bisso I, Las Heras M, Cornejo R (2024). Physiological effect of high flow oxygen therapy measured by electrical impedance tomography in single-lung transplantation. Medicina (B Aires).

[18] Shingina A, Mukhtar N, Wakim-Fleming J, Alqahtani S, Wong RJ, Limketkai BN, Larson AM, Grant L (2023). Acute liver failure guidelines. Am J Gastroenterol.

[19] Bajaj JS, O'Leary JG, Lai JC, Wong F, Long MD, Wong RJ, Kamath PS (2022). Acute-on-chronic liver failure clinical guidelines. Am J Gastroenterol.

[20] Joglar JA, Chung MK, Armbruster AL, Benjamin EJ, Chyou JY, Cronin EM (2024). 2023 ACC/AHA/ACCP/HRS guideline for the diagnosis and management of atrial fibrillation: a report of the American college of cardiology/American heart association joint committee on clinical practice guidelines. Circulation.

[21] Zeppenfeld K, Tfelt-Hansen J, de Riva M, Winkel BG, Behr ER, Blom NA (2022). 2022 ESC Guidelines for the management of patients with ventricular arrhythmias and the prevention of sudden cardiac death. Eur Heart J.

[22] Kannankeril P, Roden DM, Darbar D (2010). Drug-induced long QT syndrome. Pharmacol Rev.

[23] Said TH, Lance DW, Jeyaraj D, Fossa AA, Rosenbaum DS (2012). Transmural dispersion of repolarization as a preclinical marker of drug-induced proarrhythmia. J Cardiovasc Pharmacol.

[24] Mahida S, Hogarth AJ, Cowan C, Tayebjee MH, Graham LN, Pepper CB (2013). Genetics of congenital and drug-induced long QT syndromes: current evidence and future research perspectives. J Interv Card Electrophysiol.

[25] Kim ED, Watt J, Tereshchenko LG, Jaar BG, Sozio SM, Kao WHL, Estrella MM, Parekh RS (2019). Associations of serum and dialysate electrolytes with QT interval and prolongation in incident hemodialysis: the predictors of arrhythmic and cardiovascular risk in end-stage renal disease (PACE) study. BMC Nephrol.

[26] Yu H, Zhang L, Liu J, Liu Y, Kowey PR, Zhang Y (2017). Acquired long QT syndrome in hospitalized patients. Heart Rhythm.

[27] Tisdale JE, Jaynes HA, Kingery JR, Mourad NA, Trujillo TN, Overholser BR, Kovacs RJ (2013). Development and validation of a risk score to predict QT interval prolongation in hospitalized patients. Circ Cardiovasc Qual Outcomes.

[28] Overton K, Post JJ (2017). Association between altered QT interval in sepsis and mortality: a possible effect of antimicrobial therapy?. Intern Med J.

[29] Wasserstrum Y, Lotan D, Itelman E, Barbarova I, Kogan M, Klempfner R, Dagan A, Segal G (2016). Corrected QT interval anomalies are associated with worse prognosis among patients suffering from sepsis. Intern Med J.

[30] Barashi R, Milwidsky A, Viskin D, Giladi M, Hochstadt A, Morgan S, Rosso R, Chorin E, Viskin S (2024). Teleological reasoning for QT prolongation caused by severe bradycardia: correlation between QT interval and brain natriuretic peptide levels during atrioventricular block. Heart Rhythm.

[31] Galluzzo A, Gallo C, Battaglia A, Frea S, Canavosio FG, Botta M, Bergerone S, Gaita F (2016). Prolonged QT interval in ST-elevation myocardial infarction: predictors and prognostic value in medium-term follow-up. J Cardiovasc Med (Hagerstown).

[32] Buttà C, Zappia L, Laterra G, Roberto M (2020). Diagnostic and prognostic role of electrocardiogram in acute myocarditis: a comprehensive review. Ann Noninvasive Electrocardiol.

[33] Banai A, Szekely Y, Lupu L, Borohovitz A, Levi E, Ghantous E, Taieb P, Hochstadt A, Banai S, Topilsky Y (2022). QT interval prolongation is a novel predictor of 1-year mortality in patients With COVID-19 infection. Front Cardiovasc Med.

[34] Pool R, Gomez H, Kellum JA (2018). Mechanisms of organ dysfunction in sepsis. Crit Care Clin.

[35] Li D, Weng Y, Zhen G, Jiang L (2022). Tp-Te interval and Tp-Te/qt ratio are valuable tools in predicting poor outcome in sepsis patients. Front Cardiovasc Med.

