# Supplementary material for: Characteristics, predictors and outcomes of new-onset QT prolongation in sepsis: a multicenter retrospective study
Source: Crit Care. 2024 Apr 9;28:115. doi: 10.1186/s13054-024-04879-2 (PMC11003155; doi:10.1186/s13054-024-04879-2)
Supplement: Supplementary file 1 — Additional file 1. QT-Prolonging Medications for Exclusion in the Study. [file 13054_2024_4879_MOESM1_ESM.docx]

**QT-Prolonging Medications for Exclusion in the Study**

1. Antiarrhythmics: Amiodarone, sotalol, quinidine
2. Antibiotics: Macrolides, fluoroquinolones
3. Antidepressants: Amitriptyline, imipramine, citalopram, amitriptyline
4. Antipsychotics: Haloperidol, ziprasidone, quetiapine, thioridazine, olanzapine, risperidone
5. Others: Methadone, sumatriptan, ondansetron, cisapride
