# Supplementary material for: Characteristics, predictors and outcomes of new-onset QT prolongation in sepsis: a multicenter retrospective study
Source: Crit Care. 2024 Apr 9;28:115. doi: 10.1186/s13054-024-04879-2 (PMC11003155; doi:10.1186/s13054-024-04879-2)
Supplement: Supplementary file 2 — Additional file 2. Table S1: Predictors of 30-day mortality in patients with sepsis. [file 13054_2024_4879_MOESM2_ESM.docx]

**Supplementary Table 1 Predictors of 30-day mortality in patients with sepsis**

**（A） Univariate analysis for predictors of 30-day mortality**

|  | 30-day survival  (n=857) | 30-day mortality  (n=167) | *P* Value |
| --- | --- | --- | --- |
| **Patient baseline** |  |  |  |
| Age, years | 60.2 ± 16.2 | 63.6 ± 15.2 | **0.013** |
| Sex, female, % | 288 (33.61) | 44 (26.35) | 0.067 |
| BMI*^a^* (kg/m²) | 23.32 ± 3.52 | 22.50 ± 3.53 | **0.006** |
| Body surface area (m²) | 1.73 ± 0.18 | 1.70 ± 0.18 | 0.091 |
| **Clinical characteristics** |  |  |  |
| Hypertension, % | 256 (29.87) | 39 (23.35) | 0.089 |
| Diabetes mellitus, % | 193 (22.52) | 30 (17.96) | 0.192 |
| Coronary artery disease, % | 64 (7.47) | 17 (10.18) | 0.235 |
| Structural heart disease, % | 46 (5.37) | 22 (13.17) | **<0.001** |
| Congenital heart disease, % | 14 (1.63) | 0 (0) | 0.096 |
| Renal insufficiency, % | 43 (5.02) | 15 (8.98) | **0.043** |
| COPD*^b^*, % | 39 (4.55) | 31 (18.56) | **<0.001** |
| Liver cirrhosis, % | 26 (3.03) | 7 (4.19) | 0.438 |
| Recent*^c^* surgery, % | 134 (15.64) | 37 (22.16) | **0.039** |
| Recent*^c^* chemo/immunotherapy, % | 63 (7.35) | 7 (4.19) | 0.139 |
| Charlson score | 1 [0, 2] | 2 [1, 3] | **<0.001** |
| Septic shock, % | 147 (17.15) | 122 (73.05) | **<0.001** |
| SOFA*^d^* score | 3 [2, 6] | 9 [8, 10] | **<0.001** |
| MODS*^e^*, % | 47 (5.48) | 94 (56.29) | **<0.001** |
| Gram-negative bacteria, % | 223 (26.02) | 37 (22.16) | 0.109 |
| Non-bacteria, % | 36 (4.20) | 11 (6.59) | 0.178 |
| **Electrocardiogram** |  |  |  |
| QTP*^f^*, % | 167 (19.49) | 68 (40.72) | **<0.001** |
| AT*^g^*/AF*^h^*, % | 42 (4.90) | 25 (14.97) | **<0.001** |
| PVC*^i^*, % | 32 (3.73) | 16 (9.58) | **0.001** |
| VT*^j^*/VF*^k^*, % | 7 (0.82) | 11 (6.59) | **<0.001** |

**(B) Multivariate analysis for the relationship between ECG abnormalities and 30-day mortality**

| Characteristic | OR | 95%CI | P value |
| --- | --- | --- | --- |
| QTP*^f^* | 1.62 | 1.01-2.58 | 0.044 |
| AT*^g^*/AF*^h^* | 1.65 | 0.79-3.50 | 0.184 |
| PVC*^i^* | 0.93 | 0.39-2.21 | 0.864 |
| VT*^j^*/VF*^k^* | 1.64 | 0.53-5.07 | 0.389 |

*Adjusted for age, body mass index, recent surgery, structural heart disease, Charlson score (including COPD^b^ and Renal insufficiency),* SOFA*^d^* score

**(C) Multiple regression models to validate the predictive value of QTP for 30-day mortality**

| Models | OR | 95%CI | P value |
| --- | --- | --- | --- |
| Model 1: adjusted for age, BMI*^a^* | 2.71 | 1.90-3.87 | <0.001 |
| Model 2: adjusted for age, BMI*^a^*, Structural heart disease | 2.54 | 1.77-3.65 | <0.001 |
| Model 3: adjusted for age, BMI*^a^*, Structural heart disease, Renal insufficiency | 2.51 | 1.75-3.61 | <0.001 |
| Model 4: adjusted for age, BMI*^a^*, Structural heart disease, Renal insufficiency, COPD*^b^* | 2.26 | 1.56-3.27 | <0.001 |
| Model 5: adjusted for age, BMI*^a^*, Structural heart disease, Charlson score | 2.31 | 1.60-3.34 | <0.001 |
| Model 6: adjusted for age, BMI*^a^*, Structural heart disease, Charlson score, Recent*^c^* surgery | 2.31 | 1.60-3.34 | <0.001 |
| Model 7: adjusted for age, BMI*^a^*, Structural heart disease, Charlson score, Recent*^c^* surgery, SOFA*^d^* score | 1.73 | 1.10-2.73 | 0.019 |
| Model 8: adjusted for age, BMI*^a^*, Structural heart disease, Charlson score, Recent*^c^* surgery, SOFA*^d^* score, AT*^g^*/AF*^h^* | 1.66 | 1.05-2.64 | 0.030 |
| Model 9: adjusted for age, BMI*^a^*, Structural heart disease, Charlson score, Recent*^c^* surgery, SOFA*^d^* score, AT*^g^*/AF*^h^*, PVC*^i^* | 1.67 | 1.05-2.64 | 0.030 |
| Model 10: adjusted for age, BMI*^a^*, Structural heart disease, Charlson score, Recent*^c^* surgery, SOFA*^d^* score, AT*^g^*/AF*^h^*, PVC*^i^*, VT*^j^*/VF*^k^* | 1.62 | 1.01-2.58 | 0.044 |

1. BMI=body mass index
2. COPD=chronic obstructive pulmonary disease.
3. Within 30 days
4. SOFA=sequential organ failure assessment
5. MODS=multiple organ dysfunction syndrome
6. QTP=QT prolongation
7. AT=atrial tachyarrhythmia
8. AF=atrial fibrillation
9. PVC=premature ventricular contraction
10. VT=ventricular tachycardia
11. VF=ventricular fibrillation
