# Supplementary material for: Characteristics, predictors and outcomes of new-onset QT prolongation in sepsis: a multicenter retrospective study
Source: Crit Care. 2024 Apr 9;28:115. doi: 10.1186/s13054-024-04879-2 (PMC11003155; doi:10.1186/s13054-024-04879-2)
Supplement: Supplementary file 3 — Additional file 3. Table S2: Multiple regression models for validate the risk factors for QTP. [file 13054_2024_4879_MOESM3_ESM.docx]

**Supplementary Table 2 Multiple regression models for validate the risk factors for QTP**

**1）10 models to analyze the predictive value of coronary artery disease for QTP*^a^***

| Models | OR | 95%CI | P value |
| --- | --- | --- | --- |
| Model 1: adjusted for age, Body surface area | 2.79 | 1.73-4.49 | <0.001 |
| Model 2: adjusted for age, Body surface area, Structural heart disease, COPD*^b^*, Hypertension, Renal insufficiency, | 2.24 | 1.35-3.71 | 0.002 |
| Model 3: adjusted for age, Body surface area, Structural heart disease, Charlson score | 2.43 | 1.48-3.98 | <0.001 |
| Model 4: adjusted for age, Body surface area, Structural heart disease, Charlson score, Septic Shock | 2.69 | 1.60-4.50 | <0.001 |
| Model 5: adjusted for age, Body surface area, Structural heart disease, Charlson score, Septic Shock, Acute heart failure | 2.52 | 1.49-4.25 | 0.001 |
| Model 6: adjusted for age, Body surface area, Structural heart disease, Charlson score, Septic Shock, Acute heart failure, Acute renal failure, Acute respiratory failure, Acute liver failure | 2.72 | 1.59-4.63 | <0.001 |
| Model 7: adjusted for age, Body surface area, Structural heart disease, Charlson score, SOFA*^c^* | 2.62 | 1.58-4.33 | <0.001 |
| Model 8: adjusted for age, Body surface area, Structural heart disease, Charlson score, SOFA*^c^*, Gram-negative bacteria | 2.62 | 1.58-4.33 | <0.001 |
| Model 9: adjusted for age, Body surface area, Structural heart disease, Charlson score, SOFA*^c^*, Gram-negative bacteria, Lung infection | 2.62 | 1.58-4.33 | <0.001 |
| Model 10: adjusted for age, Body surface area, Structural heart disease, Charlson score, SOFA*^c^*, Gram-negative bacteria, Lung infection, white cell count, neutrophil, CRP^d^, albumin | 1.94 | 1.02-3.70 | 0.044 |

**2）10 models to analyze the predictive value of septic shock for QTP**

| Models | OR | 95%CI | P value |
| --- | --- | --- | --- |
| Model 1: adjusted for age, Body surface area | 3.31 | 2.42-4.53 | <0.001 |
| Model 2: adjusted for age, Body surface area, Structural heart disease, COPD*^b^*, Hypertension | 3.14 | 2.26-4.35 | <0.001 |
| Model 3: adjusted for age, Body surface area, Structural heart disease, COPD*^b^*, Hypertension, Coronary artery disease, Renal insufficiency, | 3.21 | 2.31-4.46 | <0.001 |
| Model 4: adjusted for age, Body surface area, Structural heart disease, Charlson score | 3.10 | 2.25-4.28 | <0.001 |
| Model 5: adjusted for age, Body surface area, Structural heart disease, Charlson score, Acute heart failure | 2.68 | 1.93-3.74 | <0.001 |
| Model 6: adjusted for age, Body surface area, Structural heart disease, Charlson score, Acute heart failure, Acute renal failure | 2.21 | 1.55-3.15 | <0.001 |
| Model 7: adjusted for age, Body surface area, Structural heart disease, Charlson score, Acute heart failure, Acute renal failure, Acute respiratory failure, Acute liver failure | 1.65 | 1.12-2.44 | 0.011 |
| Model 8: adjusted for age, Body surface area, Structural heart disease, Charlson score, Acute heart failure, Acute renal failure, Acute respiratory failure, Acute liver failure, Gram-negative bacteria | 1.69 | 1.15-2.50 | 0.008 |
| Model 9: adjusted for age, Body surface area, Structural heart disease, Charlson score, Acute heart failure, Acute renal failure, Acute respiratory failure, Acute liver failure, Gram-negative bacteria, Lung infection | 1.71 | 1.16-2.52 | 0.007 |
| Model 10: adjusted for age, Body surface area, Structural heart disease, Charlson score, Acute heart failure, Acute renal failure, Acute respiratory failure, Acute liver failure, Gram-negative bacteria, Lung infection, white cell count, neutrophil, CRP^d^, albumin | 1.09 | 0.64-1.86 | 0.747 |

**3）10 models to analyze the predictive value of acute heart failure for QTP**

| Models | OR | 95%CI | P value |
| --- | --- | --- | --- |
| Model 1: adjusted for age, Body surface area | 4.22 | 2.90-6.16 | <0.001 |
| Model 2: adjusted for age, Body surface area, Structural heart disease, COPD*^b^*, Hypertension | 3.52 | 2.33-5.31 | <0.001 |
| Model 3: adjusted for age, Body surface area, Structural heart disease, COPD*^b^*, Hypertension, Coronary artery disease, Renal insufficiency | 3.40 | 2.24-5.15 | <0.001 |
| Model 4: adjusted for age, Body surface area, Structural heart disease, Charlson score | 3.45 | 2.29-5.19 | <0.001 |
| Model 5: adjusted for age, Body surface area, Structural heart disease, Charlson score, Septic shock | 2.69 | 1.76-4.10 | <0.001 |
| Model 6: adjusted for age, Body surface area, Structural heart disease, Charlson score, Septic Shock, Acute renal failure | 2.24 | 1.44-3.48 | <0.001 |
| Model 7: adjusted for age, Body surface area, Structural heart disease, Charlson score, Septic Shock, Acute renal failure, Acute respiratory failure, Acute liver failure | 1.80 | 1.14-2.85 | 0.012 |
| Model 8: adjusted for age, Body surface area, Structural heart disease, Charlson score, Septic shock, Acute renal failure, Acute respiratory failure, Acute liver failure, Gram-negative bacteria | 1.82 | 1.15-2.88 | 0.011 |
| Model 9: adjusted for age, Body surface area, Structural heart disease, Charlson score, Septic shock, Acute renal failure, Acute respiratory failure, Acute liver failure, Gram-negative bacteria, Lung infection | 1.88 | 1.19-2.99 | 0.007 |
| Model 10: adjusted for age, Body surface area, Hypertension, Structural heart disease, Charlson score, Septic shock, Acute renal failure, Acute respiratory failure, Acute liver failure, Gram-negative bacteria, Lung infection, white cell count, neutrophil, CRP^d^, albumin | 2.07 | 1.13-3.77 | 0.018 |

**4）10 models to analyze the predictive value of acute renal failure for QTP**

| Models | OR | 95%CI | P value |
| --- | --- | --- | --- |
| Model 1: adjusted for age, Body surface area | 3.38 | 2.44-4.68 | <0.001 |
| Model 2: adjusted for age, Body surface area, Structural heart disease, COPD*^b^*, Hypertension | 3.20 | 2.29-4.47 | <0.001 |
| Model 3: adjusted for age, Body surface area, Structural heart disease, COPD*^b^*, Hypertension, Coronary artery disease, Renal insufficiency, | 3.28 | 2.34-4.60 | <0.001 |
| Model 4: adjusted for age, Body surface area, Structural heart disease, Charlson score | 3.08 | 2.21-4.30 | <0.001 |
| Model 5: adjusted for age, Body surface area, Structural heart disease, Charlson score, Septic shock | 2.21 | 1.54-3.17 | <0.001 |
| Model 6: adjusted for age, Body surface area, Structural heart disease, Charlson score, Septic shock, Acute heart failure | 1.86 | 1.27-2.71 | 0.001 |
| Model 7: adjusted for age, Body surface area, Structural heart disease, Charlson score, Septic shock, Acute heart failure, Acute respiratory failure, Acute liver failure | 1.64 | 1.10-2.44 | 0.015 |
| Model 8: adjusted for age, Body surface area, Structural heart disease, Charlson score, Septic shock, Acute heart failure, Acute respiratory failure, Acute liver failure, Gram-negative bacteria | 1.62 | 1.09-2.42 | 0.018 |
| Model 9: adjusted for age, Body surface area, Structural heart disease, Charlson score, Septic shock, Acute heart failure, Acute respiratory failure, Acute liver failure, Gram-negative bacteria, Lung infection | 1.62 | 1.08-2.41 | 0.019 |
| Model 10: adjusted for age, Body surface area, Hypertension, Structural heart disease, Charlson score, Septic shock, Acute heart failure, Acute respiratory failure, Acute liver failure, Gram-negative bacteria, Lung infection, white cell count, neutrophil, CRP^d^, albumin | 1.68 | 1.00-2.81 | 0.048 |

**5）10 models to analyze the predictive value of acute respiratory failure for QTP**

| Models | OR | 95%CI | P value |
| --- | --- | --- | --- |
| Model 1: adjusted for age, Body surface area | 4.84 | 3.50-6.70 | <0.001 |
| Model 2: adjusted for age, Body surface area, Structural heart disease, COPD*^b^*, Hypertension | 4.39 | 3.10-6.21 | <0.001 |
| Model 3: adjusted for age, Body surface area, Structural heart disease, COPD*^b^*, Hypertension, Coronary artery disease, Renal insufficiency, | 4.45 | 3.13-6.33 | <0.001 |
| Model 4: adjusted for age, Body surface area, Structural heart disease, Charlson score | 4.28 | 3.06-5.98 | <0.001 |
| Model 5: adjusted for age, Body surface area, Structural heart disease, Charlson score, Septic shock | 3.20 | 2.20-4.63 | <0.001 |
| Model 6: adjusted for age, Body surface area, Structural heart disease, Charlson score, Septic shock, Acute heart failure | 2.77 | 1.88-4.06 | <0.001 |
| Model 7: adjusted for age, Body surface area, Structural heart disease, Charlson score, Septic shock, Acute heart failure, Acute renal failure, Acute liver failure | 2.58 | 1.75-3.82 | <0.001 |
| Model 8: adjusted for age, Body surface area, Structural heart disease, Charlson score, Septic shock, Acute heart failure, Acute renal failure, Acute liver failure, Gram-negative bacteria | 2.62 | 1.78-3.88 | <0.001 |
| Model 9: adjusted for age, Body surface area, Structural heart disease, Charlson score, Septic shock, Acute heart failure, Acute renal failure, Acute liver failure, Gram-negative bacteria, Lung infection | 2.97 | 1.94-4.56 | <0.001 |
| Model 10: adjusted for age, Body surface area, Hypertension, Structural heart disease, Charlson score, Septic shock, Acute heart failure, Acute renal failure, Acute liver failure, Gram-negative bacteria, Lung infection, white cell count, neutrophil, CRP^d^, albumin | 2.26 | 1.30-3.93 | 0.004 |

1. QTP=QT prolongation
2. COPD=chronic obstructive pulmonary disease.
3. SOFA=sequential organ failure assessment
4. CRP=C-reaction protein
